# The CAIRR Pipeline for Submitting Standards-Compliant B and T Cell Receptor Repertoire Sequencing Studies to the National Center for Biotechnology Information Repositories

**DOI:** 10.3389/fimmu.2018.01877

**Published:** 2018-08-16

**Authors:** Syed Ahmad Chan Bukhari, Martin J. O’Connor, Marcos Martínez-Romero, Attila L. Egyedi, Debra Willrett, John Graybeal, Mark A. Musen, Florian Rubelt, Kei-Hoi Cheung, Steven H. Kleinstein

**Affiliations:** ^1^Department of Pathology, Yale School of Medicine, Yale University, New Haven, CT, United States; ^2^Stanford Center for Biomedical Informatics Research, Stanford University, Stanford, CA, United States; ^3^Department of Microbiology and Immunology, Institute for Immunity, Transplantation and Infection, Stanford University School of Medicine, Stanford, CA, United States; ^4^Department of Emergency Medicine, Yale School of Medicine, Yale University, New Haven, CT, United States; ^5^Yale Center for Medical Informatics, Yale School of Medicine, Yale University, New Haven, CT, United States; ^6^Interdepartmental Program in Computational Biology and Bioinformatics, Yale University, New Haven, CT, United States

**Keywords:** immune-repertoire sequencing, Rep-seq, antibody, B cell receptor, T cell receptor, National Center for Biotechnology Information, ontology

## Abstract

The adaptation of high-throughput sequencing to the B cell receptor and T cell receptor has made it possible to characterize the adaptive immune receptor repertoire (AIRR) at unprecedented depth. These AIRR sequencing (AIRR-seq) studies offer tremendous potential to increase the understanding of adaptive immune responses in vaccinology, infectious disease, autoimmunity, and cancer. The increasingly wide application of AIRR-seq is leading to a critical mass of studies being deposited in the public domain, offering the possibility of novel scientific insights through secondary analyses and meta-analyses. However, effective sharing of these large-scale data remains a challenge. The AIRR community has proposed minimal information about adaptive immune receptor repertoire (MiAIRR), a standard for reporting AIRR-seq studies. The MiAIRR standard has been operationalized using the National Center for Biotechnology Information (NCBI) repositories. Submissions of AIRR-seq data to the NCBI repositories typically use a combination of web-based and flat-file templates and include only a minimal amount of terminology validation. As a result, AIRR-seq studies at the NCBI are often described using inconsistent terminologies, limiting scientists’ ability to access, find, interoperate, and reuse the data sets. In order to improve metadata quality and ease submission of AIRR-seq studies to the NCBI, we have leveraged the software framework developed by the Center for Expanded Data Annotation and Retrieval (CEDAR), which develops technologies involving the use of data standards and ontologies to improve metadata quality. The resulting CEDAR-AIRR (CAIRR) pipeline enables data submitters to: (i) create web-based templates whose entries are controlled by ontology terms, (ii) generate and validate metadata, and (iii) submit the ontology-linked metadata and sequence files (FASTQ) to the NCBI BioProject, BioSample, and Sequence Read Archive databases. Overall, CAIRR provides a web-based metadata submission interface that supports compliance with the MiAIRR standard. This pipeline is available at http://cairr.miairr.org, and will facilitate the NCBI submission process and improve the metadata quality of AIRR-seq studies.

## Introduction

Recent advances in next-generation sequencing technology have made it possible to profile the adaptive immune receptor repertoire (AIRR) in exquisite detail. AIRR sequencing (AIRR-seq) (1) studies can generate tens- to hundreds-of-millions of B and T cell receptor gene rearrangements per experiment. Categorization of receptor diversity and gene segment usage, along with identification of clonal lineages and shared hypervariable region motifs provide a rich and detailed view of the adaptive immune landscape (1). Since first developed in 2009 (2, 3), AIRR-seq has been broadly applied in basic and clinical research settings. For example, it has been used to monitor immune responses to vaccines and natural infections, cancer therapies, and to track autoimmune and malignant clones over time (2, 4). Secondary analyses and meta-analyses, which combine independent AIRR-seq studies, could enhance reproducibility and facilitate new scientific discoveries provided that the AIRR-seq data adhere to the findable, accessible, interoperable, and reusable (FAIR) data principles (5).

Effective sharing of large-scale experimental data is a significant challenge. Minimal information about an adaptive immune receptor repertoire (MiAIRR) sequencing experiment (6) was proposed by the AIRR Community (7) as a standard for making AIRR-seq studies sharable. Community-accepted data standards, such as MiAIRR, lower the barriers to data sharing, as experimental results can easily be transferred without the need for lengthy and error-prone descriptions of experimental conditions. In addition, analysis software can be written once to work on all data, and the standards specify the availability of key information in a machine readable format. More broadly, the availability of common standards for AIRR-Seq studies benefits the wider immunology community, with implications for both basic research and clinical medicine.

**Figure 1:**
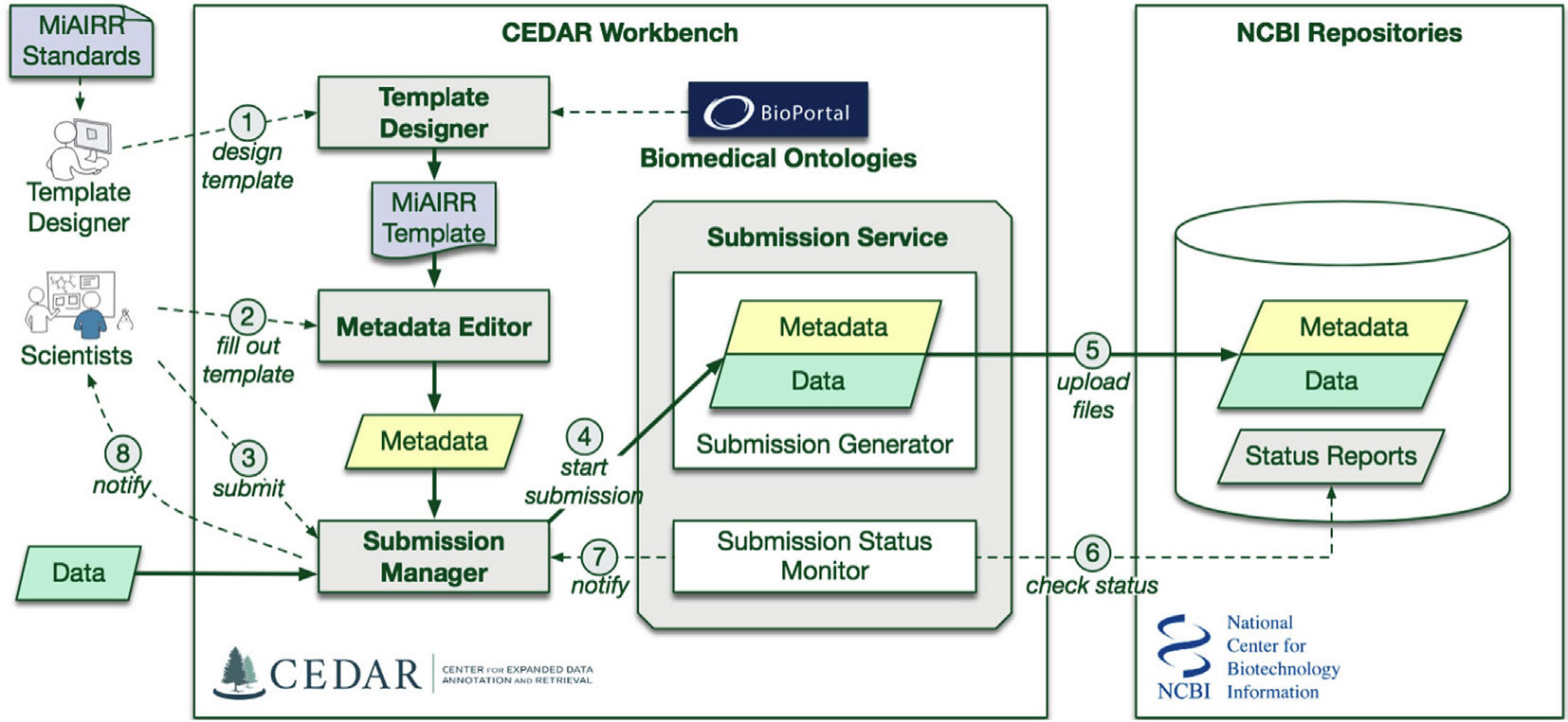
CAIRR Submission Pipeline Workflow. (1) The CEDAR Template Designer is employed to create a set of templates according to the Minimal Information about an Adaptive Immune Receptor Repertoire (MiAIRR) standard. (2) Scientists can log into the CEDAR Workbench and use these templates to edit ontology-controlled metadata associated with their AIRR-sequencing study. The edited metadata is pre-validated through the National Center for Biotechnology Information (NCBI) validation service. (3) Scientists can start the submission process by accessing the Submission Manager within their CEDAR Workbench workspace. (4) The Submission Manager connects the CEDAR Workbench to the NCBI. (5) The Submission Manager facilitates uploading the metadata and data (FASTQ files) to the NCBI. (6) The CAIRR pipeline periodically checks the submission status at the NCBI. (7) Alert messages from NCBI are received by the Submission Manager. (8) These alert messages provide step-by-step processing detail to the scientists.

We used Center for Expanded Data Annotation and Retrieval (CEDAR) technology (8) to develop a submission pipeline for AIRR-seq studies into National Center for Biotechnology Information (NCBI) repositories. Four NCBI repositories are needed to cover the full set of required MiAIRR data elements (6): BioProject, BioSample (9), the Sequence Read Archive (SRA) (10), and GenBank (11). Study, subject, and sample information is submitted to BioProject and BioSample, while the sequencing information and linked raw sequencing data are submitted to SRA. Processed sequencing data are submitted to GenBank. Submissions of AIRR-seq data to the NCBI repositories typically use a combination of web-based and flat-file templates and include only a minimal amount of terms validation. As a result, metadata at these NCBI repositories are often described using inconsistent terminologies, limiting scientists’ ability to access, find, interpret, and reuse the data sets, and to understand how the experiments were performed. Ontologies help to contextually interpret the heterogeneous metadata by associating the metadata concepts with ontology classes (12, 13). CEDAR develops technology that takes advantage of data standards and ontologies to improve metadata consistency and interoperability (8, 14, 15). We have leveraged CEDAR technology to improve metadata quality and ease the AIRR-seq study submission process by developing an AIRR-seq data submission pipeline named CEDAR-AIRR (CAIRR) (Figure 1).

CAIRR uses CEDAR technology to: (i) create web-based data submission templates whose values are mapped to ontology terms, (ii) generate and validate metadata, and (iii) submit the ontology-linked metadata and sequence files (FASTQ) (16) to the NCBI BioProject, BioSample, and SRA databases. Overall, CAIRR provides a web-based metadata submission interface that supports compliance with MiAIRR standard, with the exception of GenBank data submission (which is still in progress). The interface enables ontology-based validation for several data fields, including: organism, disease, cell type and subtype, and tissue (17). This pipeline (Figure 1) will facilitate the NCBI submission process and improve the metadata quality of AIRR-seq studies.

## Miairr-Compliant Template Development Leveraging Cedar Template Editor

**Figure 2:**
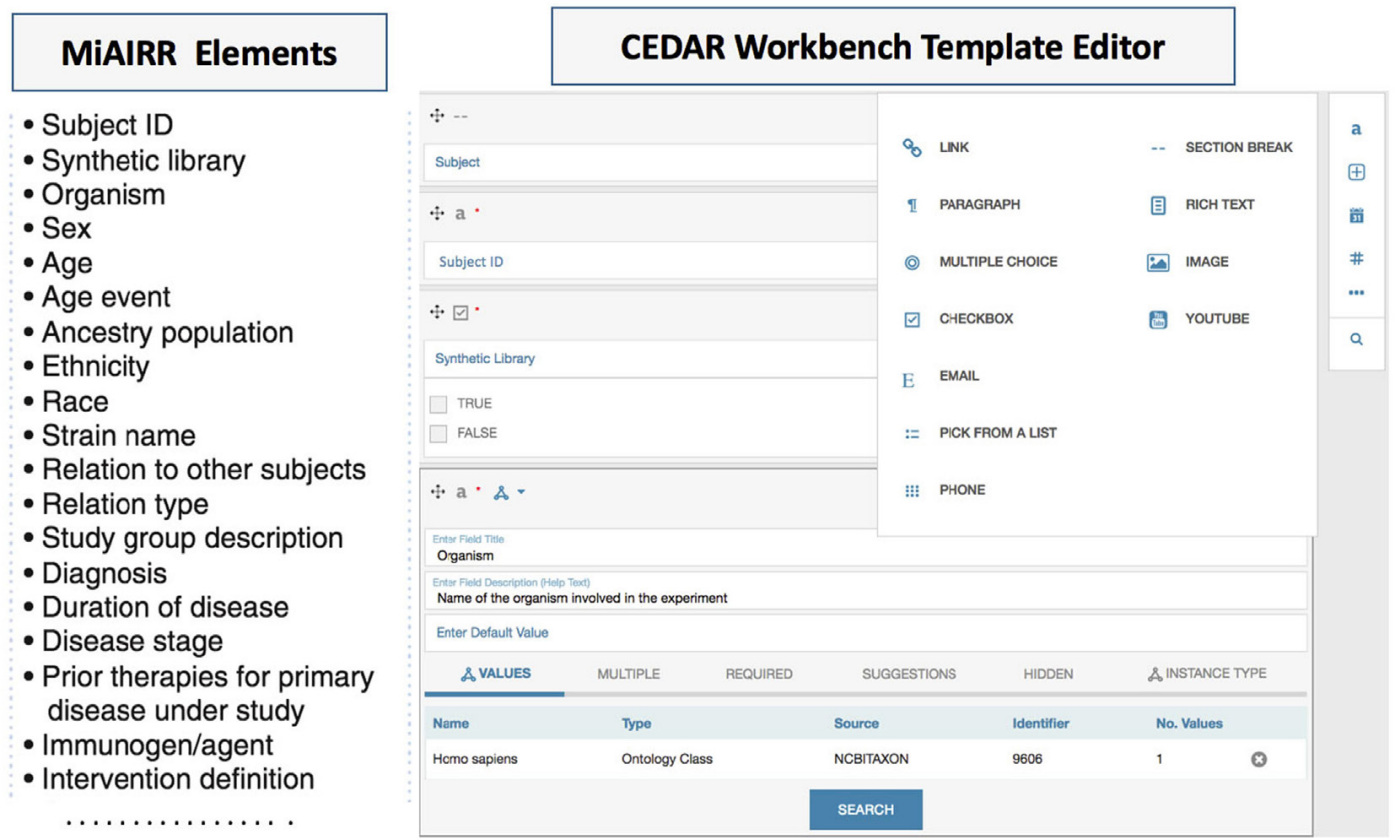
The Minimal Information about an Adaptive Immune Receptor Repertoire (MiAIRR) fields are transformed into a CEDAR template using the CEDAR Template Designer. Fields specified by MiAIRR (left panel) are transformed into a CEDAR template (right panel).

The CEDAR Workbench provides the CEDAR Template Designer, a module to create metadata templates or web forms for metadata editing. These templates consist of fields each of which contains one or more atomic pieces of information, such as a text or date field, or may be recursively composed from other template fields (Figure 2, right panel) (18). Fields can be restricted to accept certain data types (e.g., number and text) and can be configured to make them mandatory or to accept multiple values. To enrich the template fields with controlled vocabularies or ontologies, the CEDAR Template Designer provides a utility for searching and linking the ontology-controlled vocabularies from the NCBO (National Center for Biomedical Ontology) BioPortal. BioPortal is a repository for biomedical ontologies (Figure 2, organism panel view) (18, 19). Linking ontologies with template fields makes the resulting metadata interoperable, which helps to accelerate the meta-analysis process and enhances study reproducibility.

We used the CEDAR Template Designer to design metadata submission templates implementing the MiAIRR standard. To effectively share AIRR-seq studies, MiAIRR specifies a list of 82 fields (Figure 2 left panel) which are categorized into six sets: (i) study, subject, and diagnosis, (ii) sample collection, (iii) sample processing and sequencing, (iv) raw sequences, (v) data processing, and (vi) processed sequences with annotations (6). The CEDAR-based MiAIRR template currently includes the first four MiAIRR sets with 66 fields because the CAIRR pipeline is not covering the submission to GenBank yet. In addition, we have included four SRA database specific fields (library_startegy, library_source, library_layout), which are not part of MiAIRR, but are mandatory elements for the repositories (e.g., isolate, geolocation, and library information in SRA, etc.) (20). The MiAIRR elements are mapped to BioProject, BioSample, and the SRA repositories in the NCBI. Overall, we have created three templates for the BioProject, BioSample, and the SRA and then grouped them into a single template called “MiAIRR Template.”

To make an AIRR study findable, we devised a scheme to link the components (e.g., BioSample and the SRA records of an AIRR study) to each other through unique identifiers in the MiAIRR template. For example, a typical AIRR study consists of multiple BioSample and SRA records and these records should be anchored to each other in a way that a human or machine can navigate from a particular BioSample record to the related SRA record. Since each BioSample is represented with a unique identifier, we used BioSample identifier as a *prime identifier* and linked BioSample records to the related SRA records with unique BioSample identifiers. This functionality helps to reduce an AIRR study metadata creation and submission time, since users can instantiate multiple BioSample and the SRA submission without worrying how the NCBI translates the resulting AIRR study data.

Linking ontologies with template fields can help make the entered metadata interoperable. In the MiAIRR template, we have constrained the field values to ontology terms. For instance, we restricted the organism, cell type, cell subtype, disease, and tissue fields to terms from AIRR community recommended ontologies such as: National Center for Biotechnology Information Taxonomy Ontology (NCBITAXON) (21), cell ontology (CL) (22), Brenda Tissue Ontology (23), and Human Disease Ontology (DOID) (24) (note that CL covers both the cell type and cell subtype). By employing the CEDAR Template Designer module, we created a MiAIRR template to fulfill the AIRR data submission needs.

## Ontology-Controlled Metadata Editing

In the CAIRR pipeline, fields are associated with available ontologies. These associations allow CEDAR to provide autocomplete functionality using the controlled vocabularies from the linked ontologies. Moreover, CEDAR ensures that all ontology-linked field values come only from ontologies and prevents free text from being used. For instance, when a user starts typing “*Homo sapiens*” in the *organism* field, controlled metadata from the NCBITAXON ontology shows up (Figure 2) (21). This ontology-based auto-completion reduces typographical errors and promotes consistent metadata entry practices. Moreover, filling a template with ontology-linked metadata enhances the ability to carry out semantic search of the submitted studies. NCBI does not make pervasive use of controlled terms as NCBI does employ the NCBI taxonomy for the organism field but features are not still implemented for the semantic search. If semantic search interface is implemented at the NCBI, a study could be searched based on its related metadata. For example, since *Homo sapiens* is a subclass of mammalia in the ontology hierarchy of NCBITAXON, it would be possible to expand the query search scope based on parent class or to narrow down the scope of a query based on the subclasses of “*Homo sapiens*” only.

**Figure 3:**
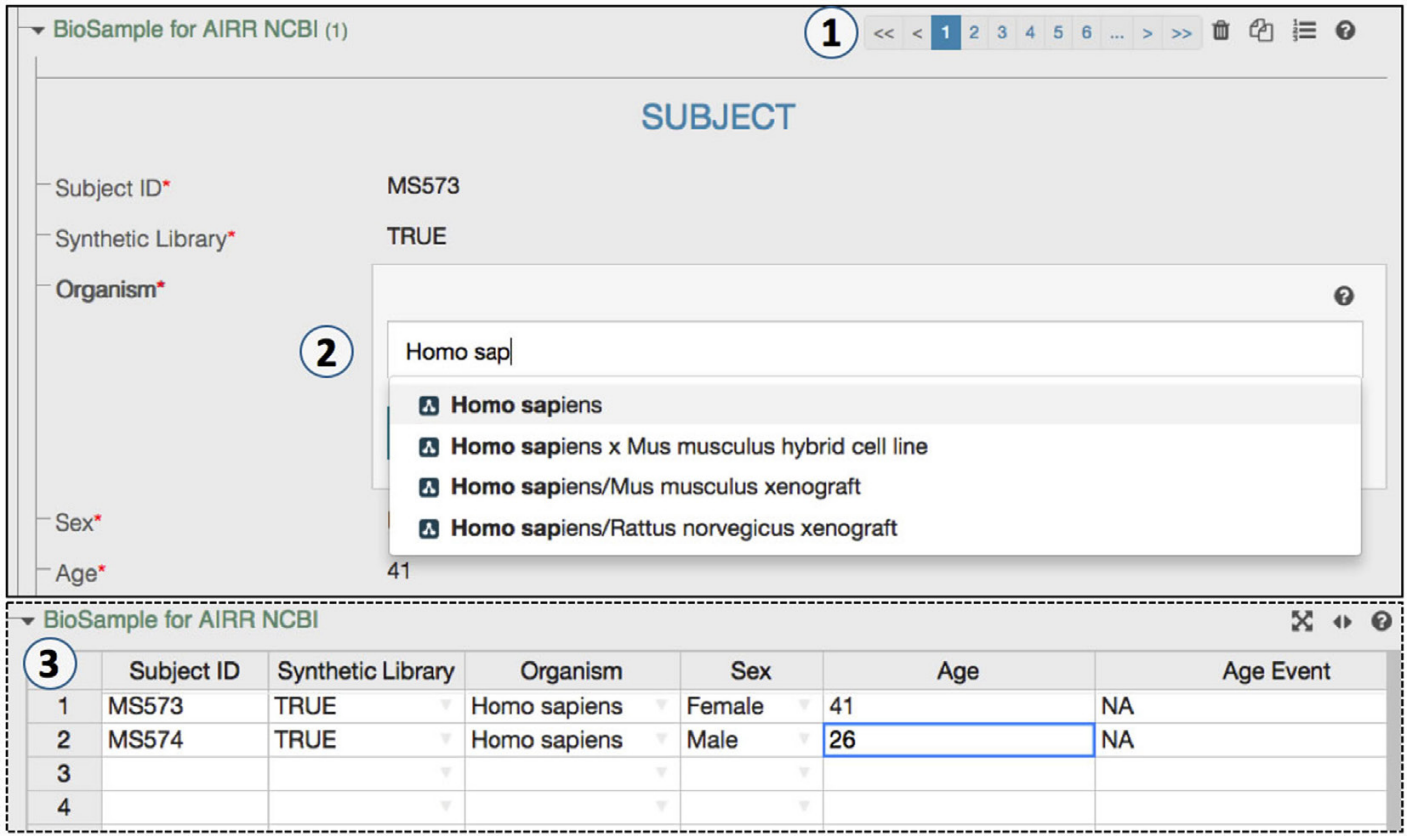
An ontology-controlled adaptive immune receptor repertoire study metadata editing process. (1) CEDAR’s Metadata Editor presents this web form based on the MiAIRR template produced by the Template Designer. The paging option allows a data submitter to add or delete BioSample and sequence read archive (SRA) records. (2) Some of BioSample and the SRA metadata are controlled through ontologies, which allow for auto-completion during data entry. (3) The toggle spreadsheet option allows data submitters to edit metadata using a traditional spreadsheet view.

The CAIRR pipeline provides a user-friendly interface for metadata creation. Features such as spreadsheet mode make the metadata editing process easy and efficient (Figure 3). For example, an AIRR study may hold multiple BioSample and SRA records, and the CAIRR pipeline allows users to add multiple records. Entering metadata into web-based templates is not always the preferred option for scientists who already have metadata available in spreadsheets (Figure 3). Therefore, we introduced a toggle spreadsheet view which works like any other traditional spreadsheet. Scientists can import existing spreadsheet hosted data into CAIRR pipeline by copying and pasting through the CAIRR spreadsheet toggle feature. Importantly, metadata validation based on ontologies and other template level constraints still works in spreadsheet view, which otherwise is not possible without writing special macros in programs like Microsoft Excel (25) or by using third-party spreadsheet ontology utilities such as RightField (26). Thus, CAIRR helps scientists to edit ontology-controlled metadata with ease and efficiency.

## Airr Study Metadata Validation and Submission

**Figure 4:**
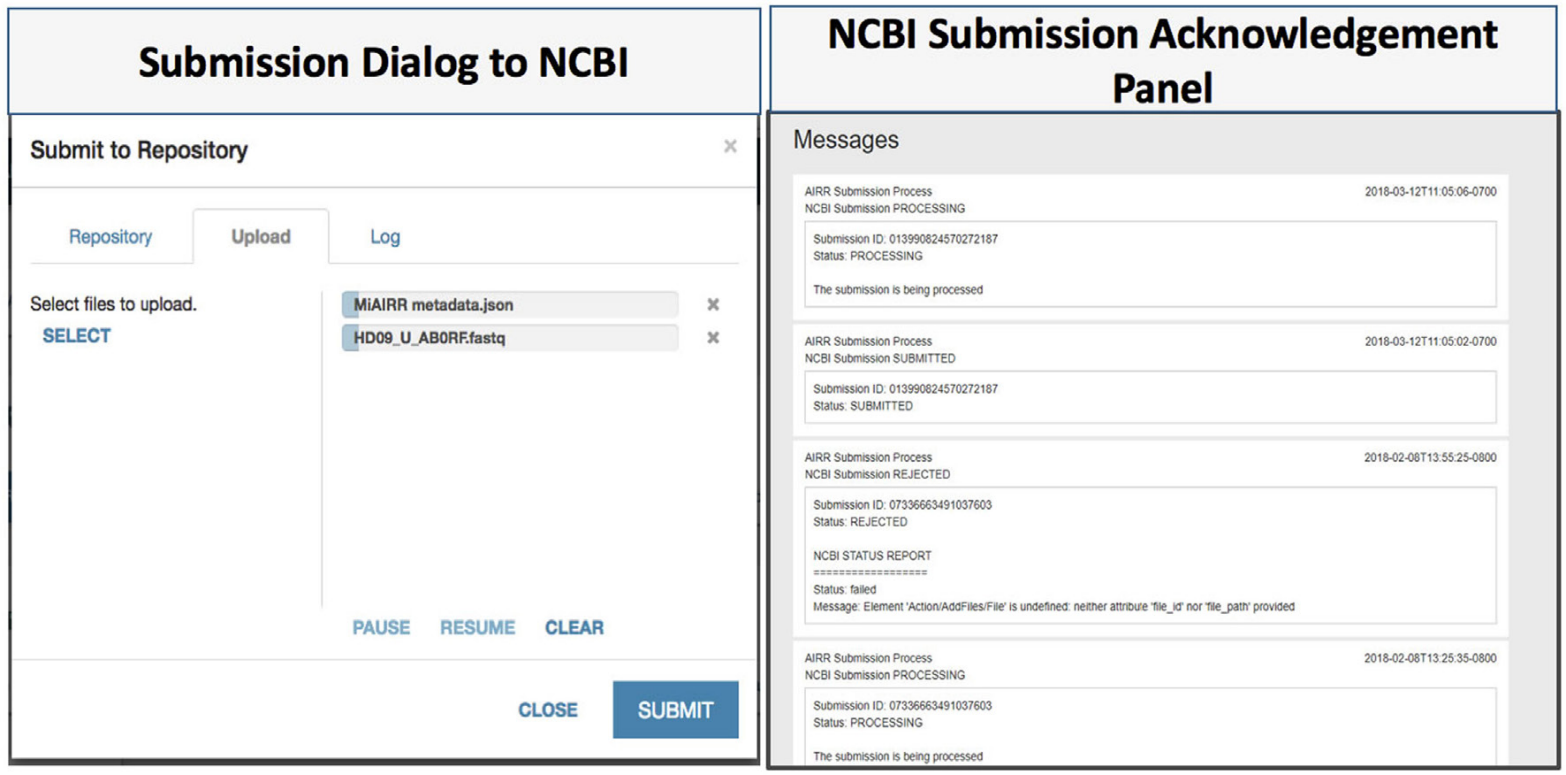
CAIRR data submission. (1) Data submitters choose National Center for Biotechnology Information (NCBI) Sequence Read Archive (SRA) as the target repository, and then upload the related datasets to submit. (2) CAIRR provides submission acknowledgment and data-processing-level messages generated by the NCBI system.

The CAIRR pipeline provides ontology-controlled suggestions at entry-time along with data type checks for the entered values (e.g., date, string, and number). To ensure the quality of the submitted metadata to the NCBI, we have designed a metadata validation module by employing the NCBI validation service which provides an additional layer of quality control (Figure 4). The NCBI validation service is publicly available for any external user or application. It detects missing mandatory BioSample fields, such as BioSample Identifier, age, isolate, and sex, and generates alerts with error messages. To use the validation service inside the CAIRR pipeline, a user fills in an AIRR study’s metadata in the MiAIRR template and invokes the validation service through the Validate Metadata option within the Metadata Editor. The validation service fetches the entered metadata and reports any non-compliant metadata. This validation service could be invoked multiple times by a data submitter during the AIRR study metadata authoring process. Thus, the CAIRR pipeline includes multi-layered validation mechanism to ensure that the submitted metadata is of a high quality and compliant with the NCBI repositories.

An AIRR study consists of AIRR metadata along with raw and processed sequence reads which are stored in FASTQ format (16). The available options for data and metadata submission using the NCBI submission interface are depositing through email or submitting through the file transfer protocol (FTP) using command line or third-party FTP utilities. In order to make the submission process easier, the CAIRR pipeline provides a user-friendly data submission interface. This data uploading facility can be accessed through the CEDAR Workspace—the first CEDAR interface users see after logging in—where users can select the generated metadata file and submit it to the NCBI repositories (Figure 4, submission dialog to the NCBI).

The CAIRR pipeline provides post-submission processing information to the submitters. Data submitters are informed within the CAIRR pipeline if any error is automatically detected after an AIRR study submission to the NCBI. The post-processing at the NCBI involves both computer-based validation and a human curator check. The computer automatically checks for the sequence reads length and its format details while a human curator looks for data relevancy and submitted metadata anomalies. Each computerized stage generates processing logs which are stored as a report. The logs capture the submitter detail, IP address, number of submitted files, and time zone information, along with the NCBI approval and rejection status information. The CAIRR pipeline parses this log file and displays the messages in the submitter’s workspace (Figure 4, NCBI submission acknowledgment panel).

## Discussion

The CAIRR pipeline was designed in compliance with the MiAIRR standard to facilitate AIRR study metadata generation and submission (see Figure S1 in Supplementary Material). In order to help users improve their metadata quality through ontology-constrained AIRR metadata selections, the CAIRR pipeline employs CEDAR technology in conjunction with NCBO BioPortal ontologies to develop the MiAIRR template. CAIRR makes AIRR study submission to the NCBI straightforward by providing a Submission Manager which handles data uploading and notifies users about post-submission processing at the NCBI. CAIRR also generates its output in JSON-LD and RDF (Resource Description Framework) formats which could be deposited into other AIRR-specific repositories such as VDJServer (27) and iReceptor (28), or into general repositories such as Zenodo.^1^

The possibility of re-analysis and meta-analysis of datasets made available through the NCBI offers the potential for important insights. However, such analyses largely depend on the effective sharing of large-scale experimental data such as that generated by AIRR sequencing studies. As next-generation sequencing technologies continue to improve, scientists are adopting these technologies to get insights into the adaptive immune response in healthy individuals and in individuals with a wide range of diseases (29, 30). The number of published and publicly available AIRR-seq datasets is also steadily increasing in repositories such as NCBI. Because metadata production is not a straightforward process, we observe some existing metadata at the NCBI with several metadata anomalies (31). The CAIRR pipeline simplifies AIRR study metadata editing and submission, thus improving the production and sharing of AIRR-seq data for further analysis.

The CAIRR pipeline can be extended in several ways. The current production version of the CAIRR pipeline supports the generation of metadata and deposition into three repositories at the NCBI (BioProject, BioSample, and the SRA). MiAIRR standard also mandates the deposition of processed data, which is not covered by these repositories. To address this, CAIRR will be extended to support submission to the NCBI GenBank. Another future extension will involve the development of an AIRR ontology, which will address the fact that not all the MiAIRR template fields are linked to ontology classes because of the unavailability of the appropriate ontology classes (e.g., forward and reverse PCR primer target locations, physical linkage of different loci). Finally, a community-level evaluation will be carried out to supplement the more limited evaluation described here.

## Conclusion

To improve AIRR study metadata quality and to facilitate the metadata creation and submission process we have developed the CAIRR pipeline^2^ using the CEDAR Workbench. By linking MiAIRR template fields with ontologies, and providing validation checks, CAIRR minimizes metadata anomalies, such as metadata inconsistency, incomplete metadata, and incorrect metadata. Through CAIRR, users can submit MiAIRR-compliant data to the NCBI BioProject, BioSample, and the SRA repositories. To promote the maximum use of CAIRR, we have created a mailing list, online documentation with step-by-step instructions^3^ along with a video tutorial. More generally, CAIRR demonstrates how the CEDAR Workbench can be tailored for metadata editing and submission according to the needs of a particular scientific community.

## Author Contributions

Study conception and design: SACB, K-HC, SHK, MC, JG, and MM. Code implementation: SACB, MC, MM-R, DW, and AE. Validated and interpreted the results: SACB, JG, FR, MC, and DW. Drafting of manuscript: SACB, SHK, and K-HC. Critical revision: MAM, MM-R, and FR. All authors read and approved the final manuscript.

## Conflict of Interest Statement

The authors declare that the research was conducted in the absence of any commercial or financial relationships that could be construed as a potential conflict of interest.

## Footnotes

^1^http://zenodo.org (Accessed: August 6, 2017).

^2^http://cairr.miairr.org (Accessed: August 6, 2017).

^3^http://cairr-docs.miairr.org/ (Accessed: August 6, 2017).
