# Supplementary material for: The CAIRR Pipeline for Submitting Standards-Compliant B and T Cell Receptor Repertoire Sequencing Studies to the National Center for Biotechnology Information Repositories
Source: Front Immunol. 2018 Aug 16;9:1877. doi: 10.3389/fimmu.2018.01877 (PMC6105692; doi:10.3389/fimmu.2018.01877)
Supplement: Supplementary file 1 [file data_sheet_1.PDF]

*Supplementary Material*

# The CAIRR pipeline for submitting standards-compliant B and T cell receptor repertoire sequencing studies to the NCBI

**Syed Ahmad Chan Bukhari[1,] Martin J. O'Connor[2], Marcos Martínez-Romero[2], Attila L. Egyedi[2], Debra Willrett[2], John Graybeal [2], Mark A. Musen[2], Florian Rubelt[3], Kei-Hoi Cheung [4,5,6*], Steven H. Kleinstein [1,6*]**

[1]Department of Pathology, Yale School of Medicine, New Haven, CT, USA, [2]Stanford Center for Biomedical Informatics Research, Stanford University, Stanford, CA, USA, [3]Department of Microbiology and Immunology and Institute for Immunity, Transplantation and Infection, Stanford University School of Medicine, Stanford, CA, USA, [4]Department of Emergency Medicine, [5]Yale Center for Medical Informatics, Yale University School of Medicine, New Haven, CT, USA.[6]Interdepartmental Program in Computational Biology and Bioinformatics, Yale University, New Haven, Connecticut 06511, USA

*co-senior authors

Please address correspondence to: steven.kleinstein@yale.edu

**1. Supplementary Figures**

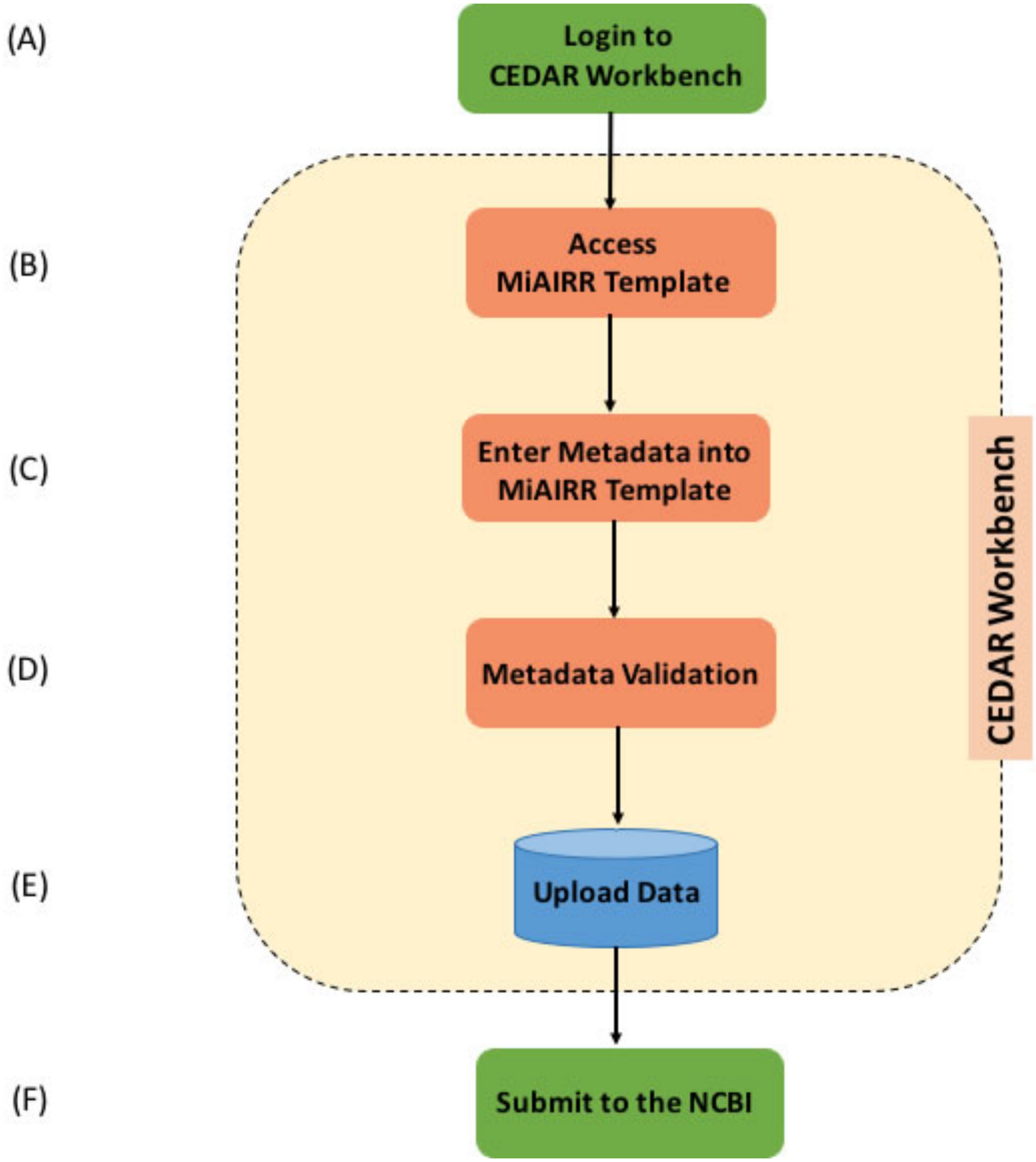


**Figure 1: Steps in AIRR-seq study submission using the CAIRR Pipeline**

(A) Access to the CAIRR pipeline is initiated by logging into the CEDAR Workbench (http://cairr.miairr.org/). The system will direct new users to a signup page, while registered users will be directed to the CEDAR Workspace after successful login. (B) The MiAIRR template is available from the CEDAR Workspace, and consists of BioProject, BioSample and the SRA sub-templates. The link in (A) provides direct access to the MiAIRR template. If using the standard CEDAR Workbench login (https://cedar.metadatacenter.org) the template can be found by searching for “MiAIRR”. Template elements can be viewed by double-clicking on the MiAIRR Template. (C) Metadata is entered by typing (or pasting) directly into the MiAIRR template. Note: For the BioProject, the current

implementation of CAIRR requires that users first obtain a BioProject ID directly from the BioProject website (https://submit.ncbi.nlm.nih.gov/subs/bioproject/). (D) To validate the entered metadata, users click on the "Validate" button. The entered metadata is then validated according to NCBI rules. (E) Sequencing data files related to the study (FASTQ or FASTA files) are uploaded for submission to SRA. (F) NCBI is selected as a target repository for data submission.
